# Data on evolutionary relationships between hearing reduction with history of disease and injuries among workers in Abadan Petroleum Refinery, Iran

**DOI:** 10.1016/j.dib.2017.12.002

**Published:** 2017-12-10

**Authors:** Mohammad Javad Mohammadi, Ebtesam Ghazlavi, Samira Rashidi Gamizji, Hajar Sharifi, Fereshteh Rashidi Gamizji, Atefeh Zahedi, Sahar Geravandi, Noorollah Tahery, Ahmad Reza Yari, Mahboobeh Momtazan

**Affiliations:** a Department of Environmental Health Engineering, School of Public Health and Environmental Technologies Research Center, Ahvaz Jundishapur University of Medical Sciences, Ahvaz, Iran; b Student Research Committee, Abadan School of Medical Sciences, Abadan, Iran; c Abadan School of Medical Sciences, Abadan, Iran; d Asadabad School of Medical Sciences, Asadabad, Iran; e Abadan School of Medical Sciences, Abadan, Iran; f Research Center for Environmental Pollutants, Qom University of Medical Sciences, Qom, Iran

**Keywords:** Abadan PR, Abadan Petroleum Refinery, HR, Hearing Reduction, Hearing reduction, Workers, Risk factors, Petroleum refinery, Iran

## Abstract

The present work examined data obtained during the analysis of Hearing Reduction (HR) of Abadan Petroleum Refinery (Abadan PR) workers of Iran with a history of disease and injuries. To this end, all workers in the refinery were chosen. In this research, the effects of history of disease and injury including trauma, electric shock, meningitis-typhoid disease and genetic illness as well as contact with lead, mercury, CO_2_ and alcohol consumption were evaluated (Lie, et al., 2016) [1]. After the completion of the questionnaires by workers, the coded data were fed into EXCELL. Statistical analysis of data was carried out, using SPSS 16.

**Specifications Table**

**Table:** 

Subject area	*Medicine, clinical research*
More specific subject area	*Risk factors accelerating hearing reduction*
Type of data	*Table, figure*
How data was acquired	*Observation hearing reduction assessment of the workers and researcher-made questionnaire analysis*
Data format	*Raw, analyzed, descriptive data*
Experimental factors	– *Sample consisted of Abadan petroleum refinery workers.* – *After selection of the workers for the study, the researcher-made questionnaires including demographic data as well as the relationship between hearing reduction with history of diseases and injuries were completed.* – *In this paper, the effects of disease and injury history including trauma, electric shock, meningitis-typhoid disease and genetic illness, as well as contact with lead, mercury, CO*_*2*_ *and alcohol consumption on accelerating hearing reduction were studied.*
Experimental features	*Hearing reduction is one of the factors which affect the efficiency of workers.*
Data source location	*Abadan, Iran*
Data accessibility	*Data are included in this article.*

**Value of the data**

• *Data describe those factors which affect the acceleration of hearing reduction among which are useful for promoting the workers' education in order to prevent this complication.*
• *Due to the importance of the relationship between hearing reduction with history of diseases and injuries among petroleum refinery workers, these factors are discussed in this article.*
• *The results showed that hearing reduction can be decreases the efficiency of workers.*
• *The results of this study can be used to develop a prevention program to decrease hearing reduction among the workers of petroleum refinery.*
• *Results are also important for workers with hearing reduction especially those workers who were detected in this study.*

## 1 Data

The dataset of this article provides information on the relationships between hearing reduction with history of disease and injuries among workers in Abadan Petroleum Refinery, Iran. Table 1 represents demographic characteristics of Abadan Petroleum Refinery workers of Iran in 2016. Table 2 shows the data of accelerating factors of hearing reduction among workers of Abadan PR.

**Table 1:** Demographic characteristics of pregnant workers in Abadan petroleum refinery.

**Parameter**	**Characteristics**	**Number (In percent)**
Gender	Male	283 (97.25%)
	Female	8 (2.75%)
Age group	15–24	19 (6.53%)
	25–34	90 (30.94%)
	35–44	125 (42.95%)
	45–55	45 (15.46%)
	More than 55	12 (4.12%)
Years of work experience	1–5 years	91(31.3%)
	5 years and more	200(68.7%)

**Table 2:** Ranking of factors affecting the accelerating hearing reduction in workers in Abadan petroleum refinery based on their importance.

**Factors**	**Number**	**Percent**
History Trauma	26	8.93
History Electric Shock	6	2.06
Meningitis-Typhoid Disease	17	5.84
Genetic Illness	12	4.12
Contact with Lead, Mercury	12	4.12
Contact with CO_2_	6	2.06
Alcohol Consumption	14	4.81

## 2 Experimental design, materials and methods

### 2.1 Description of Study area

**Fig. 1:**
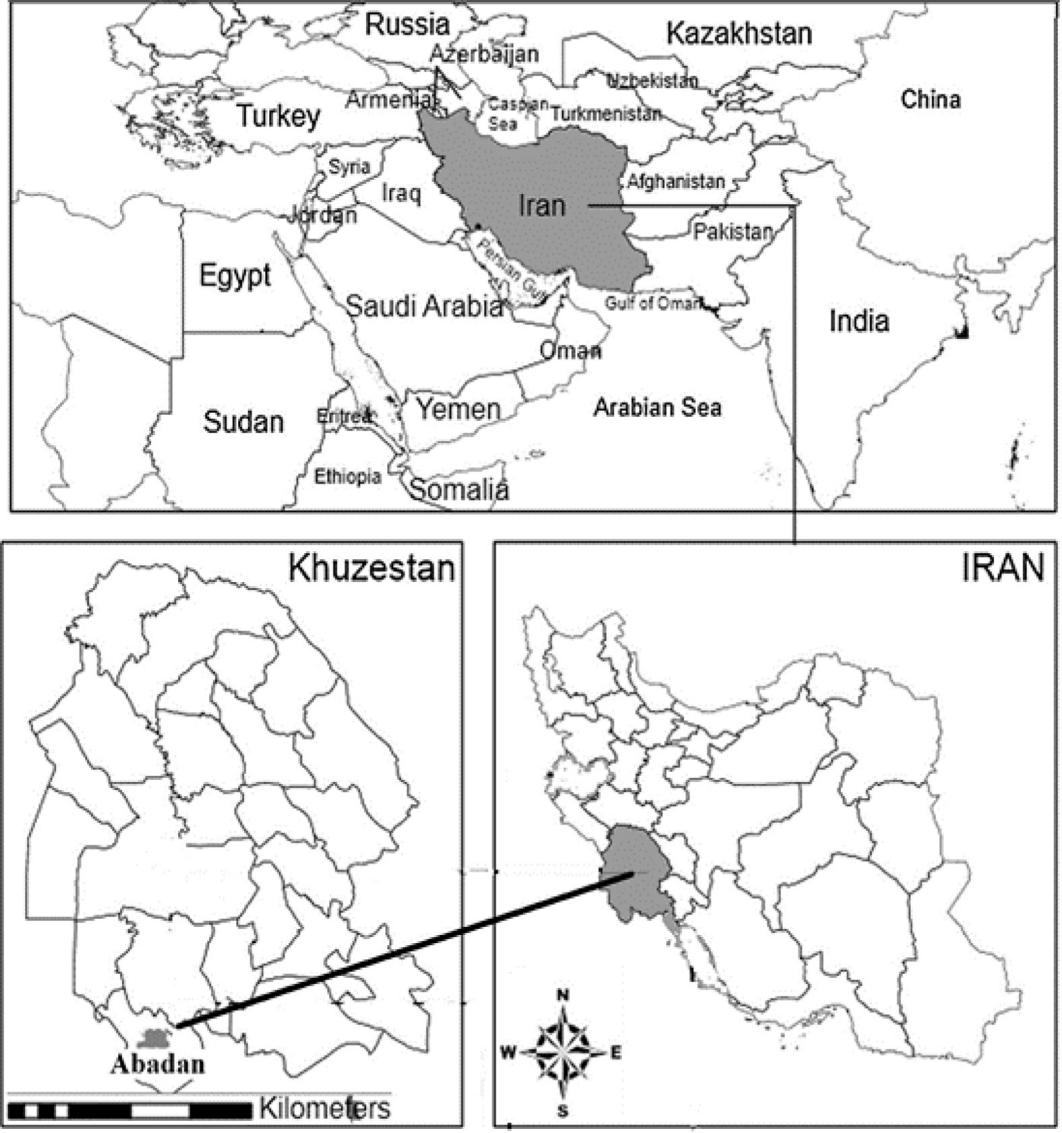
Location of Abadan in the southwest of Iran.

Petroleum Refinery of Abadan was selected for the purpose of the study. Abadan with a population of 300,000 people is one of the metropolitan cities of Khuzestan province [2]. Abadan is Located in the south of Khuzestan province, southwest of Iran (see Fig. 1). Khuzestan province has a hot and semi-humid climate with long summers and short winters [3], [4].

### 2.2 Design

291 workers of Abadan petroleum refinery participated in this study. A questionnaire was constructed by the researcher based on the risk factors adopted from Williams’ Obstetrics and Gynecology (based on the risk factors adopted from Williams’ Obstetrics and Gynecology),which comprised the demographic data (e.g. age, sex and job experience) and questions related to the causes and effective factors of the acceleration of hearing reduction, which included history of some diseases and injuries (trauma, electric shock, meningitis-typhoid disease and genetic illness), contact with lead, mercury, CO_2_ and alcohol consumption [1], [5]. After that, the collected data were coded and entered into SPSS version 16. Data analysis was performed using SPSS-16. All risk factors were taken into account. Data were analyzed using descriptive statistics.
