# Supplementary material for: Data on evolutionary relationships between hearing reduction with history of disease and injuries among workers in Abadan Petroleum Refinery, Iran
Source: Data Brief. 2017 Dec 10;16:817–21. doi: 10.1016/j.dib.2017.12.002 (PMC5773444; doi:10.1016/j.dib.2017.12.002)
Supplement: Supplementary file 1 — Supplementary material [file mmc1.doc]

**Data on evolutionary relationships between hearing reduction with history of disease and injuries among workers in Abadan Petroleum Refinery, Iran**

**Conflicts of Interest**

Authors have no conflicts of interest.

**Acknowledgment**

The authors would like to thank Student Research Committee, Abadan University of Medical Sciences for providing financial support for this research (grant No.: IR.ABADANUMS.REC.1395.109).

**Funding/Support**

The authors would like to thank Student Research Committee, Abadan University of Medical Sciences for providing financial support for this research (grant No.: IR.ABADANUMS.REC.1395.109).
